# Bis{*N*-ethyl-2-[3-(hy­droxy­imino-κ*N*)butan-2-yl­idene]hydrazinecarbothio­amide-κ^2^ *N* ^2^,*S*}nickel(II) dichloride

**DOI:** 10.1107/S1600536811055383

**Published:** 2012-01-21

**Authors:** Halema Shaban Abduelftah, Amna Qasem Ali, Naser Eltaher Eltayeb, Siang Guan Teoh, Hoong-Kun Fun

**Affiliations:** a School of Chemical Sciences, Universiti Sains Malaysia, Minden, Penang, Malaysia; b Faculty of Science, Sabha University, Libya; c Department of Chemistry, International University of Africa, Sudan; d X-ray Crystallography Unit, School of Physics, Universiti Sains Malaysia, 11800 USM, Penang, Malaysia

## Abstract

In the title complex, [Ni(C_7_H_14_N_4_OS)_2_]Cl_2_, the Ni^II^ ion is six-coordinated in a distorted octa­hedral geometry by four N atoms from the two imine and two oxime groups, and two S atoms from the thione groups. Two chloride ions complete the asymmetric unit. In the crystal, mol­ecules are linked through N—H⋯Cl and O—H⋯Cl hydrogen bonds into an infinite chain propagating along [101].

## Related literature

For bond-length data, see: Allen *et al.* (1987 ▶). For a related structure, see: Choi *et al.* (2008 ▶). For the biological activity, pharmacological properties and analytical applications of thio­semicarbazones and their metal complexes, see: Cowley *et al.* (2002 ▶); Ming (2003 ▶); Lobana *et al.* (2004 ▶, 2007 ▶).
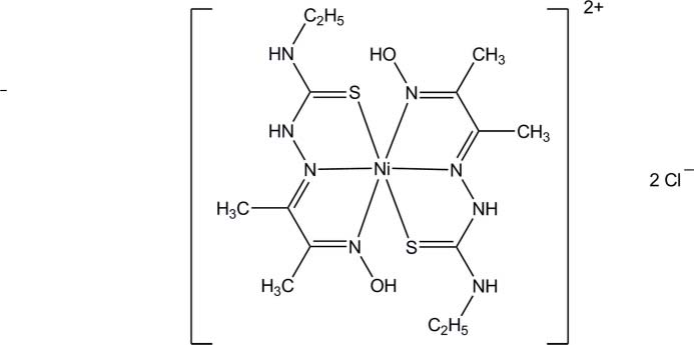


## Experimental

### 

#### Crystal data

[Ni(C_7_H_14_N_4_OS)_2_]Cl_2_
*M* *_r_* = 534.17
Monoclinic, 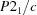

*a* = 18.4990 (11) Å
*b* = 14.2097 (9) Å
*c* = 9.2422 (6) Å
β = 98.542 (1)°
*V* = 2402.5 (3) Å^3^
*Z* = 4
Mo *K*α radiation
μ = 1.23 mm^−1^
*T* = 293 K
0.42 × 0.20 × 0.12 mm

#### Data collection

Bruker APEXII CCD diffractometer
Absorption correction: multi-scan (*SADABS*; Bruker, 2005 ▶) *T*_min_ = 0.625, *T*_max_ = 0.869
30693 measured reflections
8190 independent reflections
6071 reflections with *I* > 2σ(*I*)
*R*_int_ = 0.030

#### Refinement

*R*[*F* ^2^ > 2σ(*F* ^2^)] = 0.033
*wR*(*F* ^2^) = 0.089
*S* = 1.03
8190 reflections
292 parameters
H atoms treated by a mixture of independent and constrained refinement
Δρ_max_ = 0.66 e Å^−3^
Δρ_min_ = −0.36 e Å^−3^

### 

Data collection: *APEX2* (Bruker, 2005 ▶); cell refinement: *SAINT* (Bruker, 2005 ▶); data reduction: *SAINT*; program(s) used to solve structure: *SHELXS97* (Sheldrick, 2008 ▶); program(s) used to refine structure: *SHELXL97* (Sheldrick, 2008 ▶); molecular graphics: *SHELXTL* (Sheldrick, 2008 ▶); software used to prepare material for publication: *SHELXTL* and *PLATON* (Spek, 2009 ▶).

## Supplementary Material

Crystal structure: contains datablock(s) I, global. DOI: 10.1107/S1600536811055383/is5020sup1.cif

Structure factors: contains datablock(s) I. DOI: 10.1107/S1600536811055383/is5020Isup2.hkl

Additional supplementary materials:  crystallographic information; 3D view; checkCIF report

## Figures and Tables

**Table 1:** Selected bond lengths (Å)

Ni1—N1	2.1247 (14)
Ni1—N2	2.0120 (12)
Ni1—N5	2.1258 (13)
Ni1—N6	2.0086 (12)
Ni1—S1	2.4089 (5)
Ni1—S2	2.4126 (5)

**Table 2:** Hydrogen-bond geometry (Å, °)

*D*—H⋯*A*	*D*—H	H⋯*A*	*D*⋯*A*	*D*—H⋯*A*
N4—H1*N*4⋯Cl1^i^	0.730 (19)	2.47 (2)	3.1689 (17)	161 (2)
O2—H1*O*2⋯Cl1	0.84 (3)	2.20 (3)	3.0062 (14)	161 (2)
N7—H1*N*7⋯Cl2^ii^	0.87 (2)	2.34 (2)	3.1488 (16)	153.9 (17)
N3—H1*N*3⋯Cl1^i^	0.76 (2)	2.50 (2)	3.2015 (16)	154 (2)
O1—H1*O*1⋯Cl2	0.79 (3)	2.20 (3)	2.9396 (16)	157 (2)
N8—H1*N*8⋯Cl2^ii^	0.85 (2)	2.349 (19)	3.1567 (19)	159 (2)

Symmetry codes: (i) 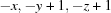; (ii) 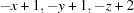.

**Fig. 1.**
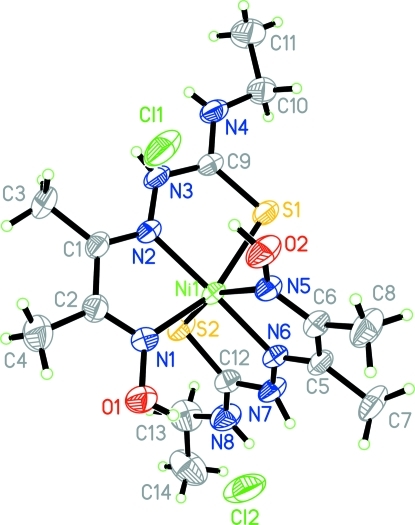
The molecular structure of the title compound, with 50% probability displacement ellipsoids and the atom-numbering scheme.

**Fig. 2.**
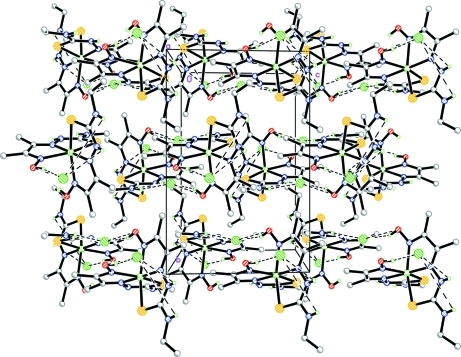
The crystal packing of the title compound viewed down the a axis. Hydrogen bonds are shown as dashed lines.

## supplementary crystallographic information

### Comment

Thiosemicarbazones and their metal complexes have attracted significant attention because of their wide-ranging biological and pharmacological properties, analytical applications, specific structures, and chemical properties (Cowley *et al.*, 2002; Ming, 2003; Lobana *et al.*, 2007; Lobana *et al.*, 2004). In this paper we report the crystal structure of bis{*N*-ethyl-2-[2-(hydroxyimino-κ*N*)butan-2-ylidene]hydrazinecarbothioamide-κ^2^*N*^2^,*S*}nickle(II)dichloride.

In the mononuclear title complex (Fig. 1), [Ni(C_7_H_14_N_4_OS)_2_]Cl_2_, the nickel(II) ion is six-coordinated in a distorted octahedral geometry by four N atoms from two imine groups and two oxime groups and two S atoms from two thione groups. The Ni—N and Ni—S bond distances (Table 1) and the bond angles around Ni1 are in agreement with the values found for related Ni(II) complex (Choi *et al.*, 2008). Bond lengths and angles observed in the structure are normal (Allen *et al.*, 1987). Ni1 is a meeting-point of four five-membered rings, namely: A (Ni1/S1/N2/N3/C9), B ((Ni1/S2/N6/N7/C12), C ((Ni1/N1/N2/C1/C2) and D ((Ni1/N5/N6/C5/C6).The dihedral angles between these four rings as follows: A/B = 87.11 (5)°, A/C = 4.37 (6)°, A/D = 88.83 (6)°, B/C = 88.55 (6)°, B/D = 4.26 (6)° and C/D = 86.88 (7)°. In the crystal, molecules are linked through intermolecular N4—H1N4···Cl1, O2—H1O2···Cl1, N7—H1N7···Cl2, N3—H1N3···Cl1, O1—H1O1···Cl2 and N8—H1N8···Cl2 hydrogen bonds (Table 2) into infinite chains propagating along [101] (Fig. 2).

### Experimental

The ligand was prepared by the mixing of 2,3-butanedione monoxime (1.01 g) dissolved in 20 ml of EtOH with 4-ethyl-3-thiosemicarbazide (1.19 g) dissolved in 20 ml of EtOH and a few drops of acetic acid. The mixture was boiled under reflux with stirring for 3 h. The mixture was filtered and left to cool and evaporate the solvent at room temperature and the resulting white solid formed was collected by suction filtration and washed with cold EtOH (yield 66%, m.p. 475.2 - 477.2 K). To a solution of the ligand (0.2021 g) in EtOH (20 ml) was added a solution of (NiCl_2_.6H_2_O) (0.2377 g) in EtOH (20 ml). The mixture was boiled under reflux for 2 h with stirring. The mixture was filtered and left to cool accompanied by slow evaporation of the solvent at room temperature. The brown crystals were grown in DMF-acetone (1:4) mixture by slow evaporation at room temperature for 2 weeks (yield 45%, m.p. 513.9 K).

### Refinement

N- and-O bound H atoms were located in a difference Fourier map and were refined freely. The remaining H atoms were positioned geometrically and refined using a riding model, with C—H = 0.96 or 0.97 Å and *U*_iso_(H) = 1.2*U*_eq_(C) for methylene groups and 1.5*U*_eq_(C) for methyl groups. The highest residual electron density peak is located 0.83 Å from Cl1 and the deepest hole is located 0.68 Å from Cl1.

### Crystal data

**Table:**

[Ni(C_7_H_14_N_4_OS)_2_]Cl_2_	*F*(000) = 1112
*M**_r_* = 534.17	*D*_x_ = 1.477 Mg m^−^^3^
Monoclinic, *P*2_1_/*c*	Mo *K*α radiation, λ = 0.71073 Å
Hall symbol: -P 2ybc	Cell parameters from 7955 reflections
*a* = 18.4990 (11) Å	θ = 2.7–31.5°
*b* = 14.2097 (9) Å	µ = 1.23 mm^−^^1^
*c* = 9.2422 (6) Å	*T* = 293 K
β = 98.542 (1)°	Block, purple
*V* = 2402.5 (3) Å^3^	0.42 × 0.20 × 0.12 mm
*Z* = 4	

### Data collection

**Table:**

Bruker APEXII CCD diffractometer	8190 independent reflections
Radiation source: fine-focus sealed tube	6071 reflections with *I* > 2σ(*I*)
graphite	*R*_int_ = 0.030
φ and ω scans	θ_max_ = 31.8°, θ_min_ = 2.7°
Absorption correction: multi-scan (*SADABS*; Bruker, 2005)	*h* = −27→24
*T*_min_ = 0.625, *T*_max_ = 0.869	*k* = −21→19
30693 measured reflections	*l* = −12→13

### Refinement

**Table:**

Refinement on *F*^2^	Primary atom site location: structure-invariant direct methods
Least-squares matrix: full	Secondary atom site location: difference Fourier map
*R*[*F*^2^ > 2σ(*F*^2^)] = 0.033	Hydrogen site location: inferred from neighbouring sites
*wR*(*F*^2^) = 0.089	H atoms treated by a mixture of independent and constrained refinement
*S* = 1.03	*w* = 1/[σ^2^(*F*_o_^2^) + (0.0413*P*)^2^ + 0.4822*P*] where *P* = (*F*_o_^2^ + 2*F*_c_^2^)/3
8190 reflections	(Δ/σ)_max_ = 0.001
292 parameters	Δρ_max_ = 0.66 e Å^−^^3^
0 restraints	Δρ_min_ = −0.36 e Å^−^^3^

### Special details

**Table:**

Geometry. All e.s.d.'s (except the e.s.d. in the dihedral angle between two l.s. planes) are estimated using the full covariance matrix. The cell e.s.d.'s are taken into account individually in the estimation of e.s.d.'s in distances, angles and torsion angles; correlations between e.s.d.'s in cell parameters are only used when they are defined by crystal symmetry. An approximate (isotropic) treatment of cell e.s.d.'s is used for estimating e.s.d.'s involving l.s. planes.
Refinement. Refinement of *F*^2^ against ALL reflections. The weighted *R*-factor *wR* and goodness of fit *S* are based on *F*^2^, conventional *R*-factors *R* are based on *F*, with *F* set to zero for negative *F*^2^. The threshold expression of *F*^2^ > σ(*F*^2^) is used only for calculating *R*-factors(gt) *etc*. and is not relevant to the choice of reflections for refinement. *R*-factors based on *F*^2^ are statistically about twice as large as those based on *F*, and *R*- factors based on ALL data will be even larger.

### Fractional atomic coordinates and isotropic or equivalent isotropic displacement parameters (Å^2^)

**Table:**

	*x*	*y*	*z*	*U*_iso_*/*U*_eq_	
Ni1	0.254705 (10)	0.544056 (14)	0.720936 (19)	0.03000 (6)	
S1	0.19095 (2)	0.69236 (3)	0.69747 (5)	0.04214 (10)	
S2	0.33886 (2)	0.59247 (4)	0.55926 (4)	0.04259 (11)	
O1	0.32326 (8)	0.33683 (10)	0.76904 (16)	0.0528 (3)	
O2	0.15128 (7)	0.47332 (11)	0.94881 (14)	0.0493 (3)	
N1	0.27241 (7)	0.39758 (10)	0.69614 (14)	0.0355 (3)	
N2	0.17389 (7)	0.50408 (10)	0.56264 (13)	0.0318 (3)	
N3	0.12478 (8)	0.56856 (10)	0.50132 (15)	0.0376 (3)	
N4	0.07395 (8)	0.71317 (11)	0.49656 (17)	0.0422 (3)	
N5	0.21534 (7)	0.51429 (10)	0.92075 (14)	0.0345 (3)	
N6	0.33784 (7)	0.58110 (9)	0.87569 (13)	0.0322 (3)	
N7	0.40248 (8)	0.61137 (11)	0.83749 (15)	0.0405 (3)	
N8	0.47653 (9)	0.63277 (13)	0.66593 (19)	0.0510 (4)	
C1	0.17121 (8)	0.41842 (12)	0.51582 (16)	0.0351 (3)	
C2	0.22784 (9)	0.35631 (12)	0.59529 (17)	0.0373 (3)	
C3	0.11569 (11)	0.38175 (14)	0.39472 (19)	0.0484 (4)	
H3A	0.1058	0.4288	0.3199	0.073*	
H3B	0.0714	0.3668	0.4324	0.073*	
H3C	0.1343	0.3261	0.3542	0.073*	
C4	0.23136 (14)	0.25422 (14)	0.5599 (3)	0.0642 (6)	
H4A	0.2650	0.2233	0.6340	0.096*	
H4B	0.2477	0.2468	0.4667	0.096*	
H4C	0.1837	0.2268	0.5563	0.096*	
C5	0.32848 (9)	0.58058 (12)	1.01105 (16)	0.0363 (3)	
C6	0.25807 (9)	0.53975 (12)	1.03697 (16)	0.0360 (3)	
C7	0.38261 (11)	0.61688 (17)	1.13467 (19)	0.0564 (5)	
H7A	0.4071	0.6708	1.1026	0.085*	
H7B	0.4178	0.5687	1.1662	0.085*	
H7C	0.3577	0.6344	1.2146	0.085*	
C8	0.24030 (12)	0.52761 (18)	1.18843 (19)	0.0585 (6)	
H8A	0.1939	0.4968	1.1841	0.088*	
H8B	0.2382	0.5882	1.2337	0.088*	
H8C	0.2775	0.4901	1.2448	0.088*	
C9	0.12641 (8)	0.65727 (11)	0.55863 (16)	0.0336 (3)	
C10	0.06787 (12)	0.81258 (14)	0.5282 (2)	0.0518 (5)	
H10A	0.1130	0.8444	0.5164	0.062*	
H10B	0.0596	0.8208	0.6286	0.062*	
C11	0.00570 (14)	0.85451 (17)	0.4263 (3)	0.0682 (6)	
H11A	0.0035	0.9210	0.4438	0.102*	
H11B	−0.0393	0.8257	0.4429	0.102*	
H11C	0.0130	0.8437	0.3270	0.102*	
C12	0.41003 (9)	0.61232 (12)	0.69315 (17)	0.0365 (3)	
C13	0.49817 (12)	0.64029 (17)	0.5214 (2)	0.0592 (5)	
H13A	0.4789	0.6980	0.4747	0.071*	
H13B	0.4783	0.5878	0.4612	0.071*	
C14	0.58014 (14)	0.64005 (19)	0.5352 (3)	0.0805 (8)	
H14A	0.5944	0.6431	0.4395	0.121*	
H14B	0.5990	0.5833	0.5831	0.121*	
H14C	0.5994	0.6935	0.5916	0.121*	
Cl1	0.03036 (3)	0.42615 (5)	0.70489 (5)	0.06167 (15)	
Cl2	0.43204 (3)	0.39179 (4)	1.01970 (8)	0.07468 (19)	
H1N4	0.0469 (11)	0.6925 (15)	0.440 (2)	0.043 (6)*	
H1O2	0.1237 (14)	0.4674 (16)	0.869 (3)	0.063 (7)*	
H1N7	0.4416 (12)	0.6072 (15)	0.903 (2)	0.050 (6)*	
H1N3	0.0897 (12)	0.5517 (14)	0.456 (2)	0.046 (6)*	
H1O1	0.3480 (13)	0.3666 (16)	0.829 (3)	0.055 (7)*	
H1N8	0.5085 (13)	0.6376 (15)	0.742 (2)	0.055 (6)*	

### Atomic displacement parameters (Å^2^)

**Table:**

	*U*^11^	*U*^22^	*U*^33^	*U*^12^	*U*^13^	*U*^23^
Ni1	0.02577 (10)	0.03547 (11)	0.02620 (9)	−0.00196 (8)	−0.00457 (6)	−0.00166 (7)
S1	0.0396 (2)	0.0390 (2)	0.0418 (2)	0.00331 (17)	−0.01353 (16)	−0.00551 (16)
S2	0.0354 (2)	0.0605 (3)	0.03009 (18)	−0.00913 (19)	−0.00085 (14)	0.00010 (17)
O1	0.0535 (8)	0.0468 (8)	0.0519 (7)	0.0120 (6)	−0.0134 (6)	−0.0010 (6)
O2	0.0352 (7)	0.0699 (9)	0.0409 (6)	−0.0168 (6)	−0.0004 (5)	0.0024 (6)
N1	0.0325 (7)	0.0383 (7)	0.0339 (6)	0.0037 (5)	−0.0007 (5)	0.0004 (5)
N2	0.0265 (6)	0.0375 (7)	0.0294 (5)	−0.0032 (5)	−0.0027 (4)	−0.0015 (5)
N3	0.0293 (7)	0.0418 (8)	0.0367 (7)	−0.0015 (6)	−0.0116 (5)	−0.0021 (5)
N4	0.0345 (8)	0.0467 (8)	0.0407 (7)	0.0033 (6)	−0.0094 (6)	0.0010 (6)
N5	0.0284 (6)	0.0406 (7)	0.0329 (6)	−0.0031 (5)	−0.0014 (5)	−0.0009 (5)
N6	0.0267 (6)	0.0373 (7)	0.0301 (6)	−0.0021 (5)	−0.0042 (4)	−0.0001 (5)
N7	0.0287 (7)	0.0552 (9)	0.0345 (6)	−0.0070 (6)	−0.0062 (5)	0.0000 (6)
N8	0.0317 (8)	0.0696 (11)	0.0509 (9)	−0.0086 (7)	0.0035 (6)	0.0000 (8)
C1	0.0299 (8)	0.0434 (9)	0.0310 (7)	−0.0063 (6)	0.0010 (5)	−0.0059 (6)
C2	0.0375 (8)	0.0375 (8)	0.0363 (7)	−0.0027 (7)	0.0037 (6)	−0.0039 (6)
C3	0.0464 (10)	0.0529 (11)	0.0416 (8)	−0.0103 (8)	−0.0073 (7)	−0.0127 (8)
C4	0.0733 (15)	0.0421 (11)	0.0715 (14)	0.0015 (10)	−0.0082 (11)	−0.0138 (10)
C5	0.0341 (8)	0.0420 (9)	0.0293 (6)	−0.0019 (7)	−0.0070 (6)	−0.0018 (6)
C6	0.0366 (8)	0.0408 (8)	0.0287 (6)	−0.0006 (7)	−0.0017 (6)	0.0004 (6)
C7	0.0516 (11)	0.0787 (14)	0.0341 (8)	−0.0182 (10)	−0.0090 (7)	−0.0095 (9)
C8	0.0573 (12)	0.0867 (16)	0.0300 (8)	−0.0173 (11)	0.0020 (7)	0.0016 (9)
C9	0.0272 (7)	0.0411 (8)	0.0306 (6)	−0.0015 (6)	−0.0018 (5)	0.0019 (6)
C10	0.0532 (11)	0.0482 (11)	0.0497 (10)	0.0130 (9)	−0.0064 (8)	0.0007 (8)
C11	0.0692 (15)	0.0600 (13)	0.0677 (13)	0.0232 (11)	−0.0153 (11)	0.0093 (11)
C12	0.0301 (8)	0.0401 (8)	0.0379 (7)	−0.0024 (6)	0.0001 (6)	0.0004 (6)
C13	0.0509 (12)	0.0671 (13)	0.0637 (12)	−0.0074 (10)	0.0218 (10)	0.0040 (10)
C14	0.0590 (15)	0.0699 (16)	0.122 (2)	−0.0031 (12)	0.0435 (15)	−0.0051 (15)
Cl1	0.0405 (3)	0.0975 (4)	0.0438 (2)	−0.0219 (3)	−0.00425 (18)	−0.0083 (2)
Cl2	0.0534 (3)	0.0625 (3)	0.0937 (4)	−0.0016 (3)	−0.0363 (3)	0.0016 (3)

### Geometric parameters (Å, °)

**Table:**

Ni1—N1	2.1247 (14)	C1—C3	1.496 (2)
Ni1—N2	2.0120 (12)	C2—C4	1.491 (3)
Ni1—N5	2.1258 (13)	C3—H3A	0.9600
Ni1—N6	2.0086 (12)	C3—H3B	0.9600
Ni1—S1	2.4089 (5)	C3—H3C	0.9600
Ni1—S2	2.4126 (5)	C4—H4A	0.9600
S1—C9	1.6927 (15)	C4—H4B	0.9600
S2—C12	1.6912 (16)	C4—H4C	0.9600
O1—N1	1.3769 (18)	C5—C6	1.478 (2)
O1—H1O1	0.79 (2)	C5—C7	1.495 (2)
O2—N5	1.3791 (18)	C6—C8	1.495 (2)
O2—H1O2	0.83 (2)	C7—H7A	0.9600
N1—C2	1.290 (2)	C7—H7B	0.9600
N2—C1	1.290 (2)	C7—H7C	0.9600
N2—N3	1.3538 (19)	C8—H8A	0.9600
N3—C9	1.366 (2)	C8—H8B	0.9600
N3—H1N3	0.76 (2)	C8—H8C	0.9600
N4—C9	1.318 (2)	C10—C11	1.497 (3)
N4—C10	1.450 (3)	C10—H10A	0.9700
N4—H1N4	0.73 (2)	C10—H10B	0.9700
N5—C6	1.2873 (19)	C11—H11A	0.9600
N6—C5	1.288 (2)	C11—H11B	0.9600
N6—N7	1.3656 (19)	C11—H11C	0.9600
N7—C12	1.362 (2)	C13—C14	1.503 (3)
N7—H1N7	0.87 (2)	C13—H13A	0.9700
N8—C12	1.324 (2)	C13—H13B	0.9700
N8—C13	1.454 (3)	C14—H14A	0.9600
N8—H1N8	0.85 (2)	C14—H14B	0.9600
C1—C2	1.479 (2)	C14—H14C	0.9600
			
N6—Ni1—N2	177.98 (5)	C2—C4—H4A	109.5
N6—Ni1—N1	102.68 (5)	C2—C4—H4B	109.5
N2—Ni1—N1	75.81 (5)	H4A—C4—H4B	109.5
N6—Ni1—N5	76.02 (5)	C2—C4—H4C	109.5
N2—Ni1—N5	105.19 (5)	H4A—C4—H4C	109.5
N1—Ni1—N5	88.68 (5)	H4B—C4—H4C	109.5
N6—Ni1—S1	98.49 (4)	N6—C5—C6	114.12 (13)
N2—Ni1—S1	83.14 (4)	N6—C5—C7	124.67 (16)
N1—Ni1—S1	158.21 (4)	C6—C5—C7	121.21 (14)
N5—Ni1—S1	91.49 (4)	N5—C6—C5	114.95 (13)
N6—Ni1—S2	82.53 (4)	N5—C6—C8	123.77 (16)
N2—Ni1—S2	96.22 (4)	C5—C6—C8	121.26 (14)
N1—Ni1—S2	95.08 (4)	C5—C7—H7A	109.5
N5—Ni1—S2	158.53 (4)	C5—C7—H7B	109.5
S1—Ni1—S2	92.701 (19)	H7A—C7—H7B	109.5
C9—S1—Ni1	95.24 (6)	C5—C7—H7C	109.5
C12—S2—Ni1	95.63 (6)	H7A—C7—H7C	109.5
N1—O1—H1O1	107.0 (17)	H7B—C7—H7C	109.5
N5—O2—H1O2	107.9 (17)	C6—C8—H8A	109.5
C2—N1—O1	112.67 (14)	C6—C8—H8B	109.5
C2—N1—Ni1	115.53 (11)	H8A—C8—H8B	109.5
O1—N1—Ni1	131.80 (10)	C6—C8—H8C	109.5
C1—N2—N3	120.53 (13)	H8A—C8—H8C	109.5
C1—N2—Ni1	119.84 (11)	H8B—C8—H8C	109.5
N3—N2—Ni1	119.56 (10)	N4—C9—N3	114.52 (14)
N2—N3—C9	119.17 (12)	N4—C9—S1	122.91 (13)
N2—N3—H1N3	118.9 (16)	N3—C9—S1	122.57 (12)
C9—N3—H1N3	118.3 (16)	N4—C10—C11	109.65 (17)
C9—N4—C10	125.00 (15)	N4—C10—H10A	109.7
C9—N4—H1N4	116.9 (17)	C11—C10—H10A	109.7
C10—N4—H1N4	118.1 (17)	N4—C10—H10B	109.7
C6—N5—O2	113.58 (13)	C11—C10—H10B	109.7
C6—N5—Ni1	114.95 (11)	H10A—C10—H10B	108.2
O2—N5—Ni1	131.47 (10)	C10—C11—H11A	109.5
C5—N6—N7	120.14 (13)	C10—C11—H11B	109.5
C5—N6—Ni1	119.40 (11)	H11A—C11—H11B	109.5
N7—N6—Ni1	120.38 (9)	C10—C11—H11C	109.5
C12—N7—N6	118.52 (13)	H11A—C11—H11C	109.5
C12—N7—H1N7	118.8 (14)	H11B—C11—H11C	109.5
N6—N7—H1N7	118.0 (14)	N8—C12—N7	114.94 (15)
C12—N8—C13	125.52 (17)	N8—C12—S2	122.81 (13)
C12—N8—H1N8	114.4 (15)	N7—C12—S2	122.22 (12)
C13—N8—H1N8	119.9 (15)	N8—C13—C14	109.5 (2)
N2—C1—C2	114.04 (13)	N8—C13—H13A	109.8
N2—C1—C3	124.57 (15)	C14—C13—H13A	109.8
C2—C1—C3	121.38 (15)	N8—C13—H13B	109.8
N1—C2—C1	114.65 (14)	C14—C13—H13B	109.8
N1—C2—C4	123.84 (17)	H13A—C13—H13B	108.2
C1—C2—C4	121.51 (15)	C13—C14—H14A	109.5
C1—C3—H3A	109.5	C13—C14—H14B	109.5
C1—C3—H3B	109.5	H14A—C14—H14B	109.5
H3A—C3—H3B	109.5	C13—C14—H14C	109.5
C1—C3—H3C	109.5	H14A—C14—H14C	109.5
H3A—C3—H3C	109.5	H14B—C14—H14C	109.5
H3B—C3—H3C	109.5		
			
N6—Ni1—S1—C9	176.16 (7)	N1—Ni1—N6—N7	91.00 (12)
N2—Ni1—S1—C9	−2.63 (6)	N5—Ni1—N6—N7	176.39 (13)
N1—Ni1—S1—C9	−17.59 (12)	S1—Ni1—N6—N7	−94.19 (12)
N5—Ni1—S1—C9	−107.75 (6)	S2—Ni1—N6—N7	−2.56 (11)
S2—Ni1—S1—C9	93.31 (6)	C5—N6—N7—C12	−179.00 (16)
N6—Ni1—S2—C12	5.33 (7)	Ni1—N6—N7—C12	−2.5 (2)
N2—Ni1—S2—C12	−173.07 (7)	N3—N2—C1—C2	−179.18 (14)
N1—Ni1—S2—C12	−96.83 (7)	Ni1—N2—C1—C2	4.00 (18)
N5—Ni1—S2—C12	2.54 (13)	N3—N2—C1—C3	−0.5 (2)
S1—Ni1—S2—C12	103.55 (6)	Ni1—N2—C1—C3	−177.35 (13)
N6—Ni1—N1—C2	−176.40 (12)	O1—N1—C2—C1	178.83 (13)
N2—Ni1—N1—C2	2.22 (11)	Ni1—N1—C2—C1	−0.92 (18)
N5—Ni1—N1—C2	108.25 (12)	O1—N1—C2—C4	−0.7 (3)
S1—Ni1—N1—C2	17.54 (19)	Ni1—N1—C2—C4	179.55 (16)
S2—Ni1—N1—C2	−92.93 (12)	N2—C1—C2—N1	−1.9 (2)
N6—Ni1—N1—O1	3.91 (15)	C3—C1—C2—N1	179.44 (15)
N2—Ni1—N1—O1	−177.48 (15)	N2—C1—C2—C4	177.68 (18)
N5—Ni1—N1—O1	−71.44 (15)	C3—C1—C2—C4	−1.0 (3)
S1—Ni1—N1—O1	−162.15 (10)	N7—N6—C5—C6	−175.83 (14)
S2—Ni1—N1—O1	87.38 (14)	Ni1—N6—C5—C6	7.6 (2)
N1—Ni1—N2—C1	−3.45 (12)	N7—N6—C5—C7	3.9 (3)
N5—Ni1—N2—C1	−88.10 (12)	Ni1—N6—C5—C7	−172.64 (15)
S1—Ni1—N2—C1	−177.77 (12)	O2—N5—C6—C5	177.75 (14)
S2—Ni1—N2—C1	90.24 (12)	Ni1—N5—C6—C5	−3.03 (19)
N1—Ni1—N2—N3	179.70 (12)	O2—N5—C6—C8	−0.7 (2)
N5—Ni1—N2—N3	95.05 (12)	Ni1—N5—C6—C8	178.50 (15)
S1—Ni1—N2—N3	5.37 (11)	N6—C5—C6—N5	−2.7 (2)
S2—Ni1—N2—N3	−86.61 (11)	C7—C5—C6—N5	177.58 (17)
C1—N2—N3—C9	176.31 (14)	N6—C5—C6—C8	175.84 (17)
Ni1—N2—N3—C9	−6.9 (2)	C7—C5—C6—C8	−3.9 (3)
N6—Ni1—N5—C6	5.21 (12)	C10—N4—C9—N3	−173.91 (17)
N2—Ni1—N5—C6	−176.46 (12)	C10—N4—C9—S1	5.8 (3)
N1—Ni1—N5—C6	108.63 (12)	N2—N3—C9—N4	−176.40 (14)
S1—Ni1—N5—C6	−93.17 (12)	N2—N3—C9—S1	3.9 (2)
S2—Ni1—N5—C6	8.1 (2)	Ni1—S1—C9—N4	−179.39 (14)
N6—Ni1—N5—O2	−175.74 (15)	Ni1—S1—C9—N3	0.27 (14)
N2—Ni1—N5—O2	2.59 (15)	C9—N4—C10—C11	174.47 (19)
N1—Ni1—N5—O2	−72.32 (14)	C13—N8—C12—N7	−178.54 (19)
S1—Ni1—N5—O2	85.88 (14)	C13—N8—C12—S2	−0.6 (3)
S2—Ni1—N5—O2	−172.89 (10)	N6—N7—C12—N8	−173.06 (16)
N1—Ni1—N6—C5	−92.46 (13)	N6—N7—C12—S2	8.9 (2)
N5—Ni1—N6—C5	−7.07 (12)	Ni1—S2—C12—N8	173.07 (15)
S1—Ni1—N6—C5	82.35 (13)	Ni1—S2—C12—N7	−9.10 (15)
S2—Ni1—N6—C5	173.99 (13)	C12—N8—C13—C14	−165.8 (2)

### Hydrogen-bond geometry (Å, °)

**Table:**

*D*—H···*A*	*D*—H	H···*A*	*D*···*A*	*D*—H···*A*
N4—H1N4···Cl1^i^	0.730 (19)	2.47 (2)	3.1689 (17)	161 (2)
O2—H1O2···Cl1	0.84 (3)	2.20 (3)	3.0062 (14)	161 (2)
N7—H1N7···Cl2^ii^	0.87 (2)	2.34 (2)	3.1488 (16)	153.9 (17)
N3—H1N3···Cl1^i^	0.76 (2)	2.50 (2)	3.2015 (16)	154 (2)
O1—H1O1···Cl2	0.79 (3)	2.20 (3)	2.9396 (16)	157 (2)
N8—H1N8···Cl2^ii^	0.85 (2)	2.349 (19)	3.1567 (19)	159 (2)

Symmetry codes: (i) −*x*, −*y*+1, −*z*+1; (ii) −*x*+1, −*y*+1, −*z*+2.
